# Impact of Transtheoretical Model Staging on Health Outcomes in Japanese Men Aged 40-70: A Propensity Score Matching Analysis

**DOI:** 10.31662/jmaj.2024-0429

**Published:** 2025-09-12

**Authors:** Rei Wakayama, Akihiko Narisada, Kohta Suzuki

**Affiliations:** 1 Department of Health and Psychosocial Medicine, Aichi Medical University School of Medicine, Aichi, Japan; 2 School of Nursing & Health, Aichi Prefectural University, Aichi, Japan

**Keywords:** transtheoretical model, behavioral health assessment, middle-aged men health, lifestyle modification, health behavior study

## Abstract

**Introduction:**

Japan’s aging population and rising healthcare costs have prompted initiatives like the National Health Program to promote preventive care. The transtheoretical model (TTM) assesses readiness for behavior change, focusing on diet and exercise. This study compared health behaviors and outcomes between Japanese men in the “precontemplation” stage (no intention to change) and those in the “contemplation or higher” stage over four years.

**Methods:**

This retrospective study analyzed data from 10,812 men aged 40-70 using the 2013-2017 Specific Health Checkups. Participants were grouped by TTM stages and matched by propensity scores to minimize confounding. Outcomes, including body mass index (BMI), blood pressure, and self-reported behaviors, were compared using χ^2^ and *t*-tests.

**Results:**

At baseline, precontemplation-stage men had better health metrics but exhibited higher rates of unhealthy behaviors, such as smoking. After four years, significant differences were observed only in high-density lipoprotein (HDL) and creatinine levels for men under 49, and in HDL, BMI, dyslipidemia medication, and eating speed for those 49 or older (p < 0.05).

**Conclusions:**

Men in the precontemplation stage face challenges related to health awareness and readiness for change. Tailored interventions, including health education and motivational interviewing, support long-term health improvements. Future research should explore personalized interventions and broader health determinants for sustained behavior change.

## Introduction

Japan’s healthcare system is grappling with rising medical expenditures, driven by a declining birth rate and an aging population. The Ministry of Health, Labor and Welfare launched the National Health Program in 2008 to address these issues and mitigate escalating social security costs, including healthcare expenses. This initiative targets individuals aged 40-74 through a combination of Specific Health Checkups and tailored health guidance to identify those at risk of lifestyle-related diseases, particularly metabolic syndrome. By prioritizing prevention and early intervention, the program seeks to avert costly conditions such as myocardial infarction and stroke, thereby alleviating the economic burden on the healthcare system ^(1)^.

As part of the Specific Health Checkup program, participants’ readiness to change lifestyle habits, such as diet and physical activity, is assessed using a question based on the transtheoretical model (TTM). The TTM delineates five stages of behavioral change: precontemplation, contemplation, preparation, action, and maintenance ^(2)^. This model supports healthcare practitioners in tailoring interventions to individuals’ stage of readiness for change while acknowledging that relapse is a common and natural part of the behavior change process ^(3)^. Ministry of Health, Labor and Welfare data reveal that 16.5% of men and 10.7% of women expressed no intention to improve unhealthy eating habits. Additionally, 13.9% of men and 11.1% of women indicated no plans to engage in physical activity ^(4)^. These figures highlight the prevalence of individuals in the precontemplation stage, who are indifferent or unmotivated to adopt healthier behaviors.

To address these challenges, the Japanese government introduced the “National Plan for the Extension of Healthy Life Expectancy” in 2019, aiming to increase healthy life expectancy by at least three years by 2040 ^(5)^. In 2019, Japan’s healthy life expectancy stood at 72.68 years for men and 75.38 years for women, compared to the national average life expectancy of 81.41 years for men and 87.45 years for women ^(6)^. Men not only have shorter overall life expectancies but also shorter healthy life expectancies than women, underscoring the need for targeted interventions to support healthier behaviors among men. Promoting men’s engagement in preventive health behaviors is essential for improving their quality of life and addressing broader societal and economic challenges linked to their shorter lifespans. This plan supports health improvement, including for individuals indifferent to health behaviors, by leveraging behavioral economics and incentives to foster behavior change and creating environments that support natural health promotion ^(5)^.

The TTM was initially applied to understand and promote smoking cessation behaviors, forming the foundation for stage-based interventions ^(7)^. However, a systematic review found limited evidence supporting the effectiveness of these interventions for smoking cessation ^(8)^. In contrast, for hypertension management, stage-based interventions grounded in the TTM framework have demonstrated promise, suggesting potential improvements in patients’ blood pressure control and related self-care behaviors ^(9)^. Additionally, observational studies have suggested that advancements in stages of change are associated with reduced risks of chronic kidney disease (CKD) and proteinuria after one year of follow-up ^(10)^. Similarly, baseline intentions to change behaviors, as assessed through the TTM, have been linked to improvements in unhealthy behaviors associated with increased cardiovascular disease (CVD) risk, including cigarette smoking, physical inactivity, skipping breakfast, and poor sleep quality at one- and two-year follow-ups ^(11)^. These findings suggest that motivation to adopt healthier behaviors, such as increasing physical activity and improving diet, could support lifestyle modifications and reduce CKD and CVD risk.

Nevertheless, the observational nature of these studies presents challenges, as unmeasured confounders may bias findings despite adjustments for known variables. Although previous studies attempted to adjust for confounding factors, residual confounding cannot be ruled out ^(10), (11)^. Furthermore, regression to the mean―a statistical phenomenon where extreme values trend toward the average with repeated measurements―complicates the interpretation of outcomes, as individuals at higher stages might naturally show better results over time. This limitation is well documented in the literature on health-related behavior changes and the TTM framework ^(12)^. Additionally, because behavior change is influenced by various psychological and environmental factors, and many of its health benefits accrue over time, long-term follow-up is essential to capture the sustained effects of behavioral intention ^(13)^. Based on the TTM, we hypothesized that individuals in the precontemplation stage at baseline would be less likely to adopt healthier behaviors or improve health status over four years than those in the contemplation or higher stages. To examine this hypothesis, we conducted a four-year follow-up study using propensity score matching (PSM), considering limitations identified in previous studies, such as short follow-up durations and potential confounding ^(14)^. This study focused on middle-aged and older Japanese men, a population characterized by lower engagement in preventive health behaviors and shorter life expectancy, to provide insights that may inform future health promotion strategies in Japan.

## Materials and Methods

### Data source

This retrospective observational study used data from the Annual Specific Health Checkups conducted by the Aichi Health Promotion Foundation, a Japanese health screening organization, from April 1, 2013, to March 31, 2018. The checkups included measurements of body mass index (BMI, calculated as weight in kilograms divided by height in square meters), waist circumference, blood tests, and questionnaires on medications and lifestyle behaviors, in accordance with the Specific Health Checkup and the Japanese Industrial Safety and Health Act ^(1)^. All examinations adhered to Japanese government regulations. This study received approval from the Institutional Review Board of Aichi Medical University School of Medicine (approval number: 18-M005). Informed consent was waived due to the anonymous nature of the data.

According to the *Aichi Prefectural Statistical Yearbook* (FY2014), the total population of Aichi Prefecture in 2013 was 7,434,996, including 3,714,009 men. Among them, 1,526,910 were aged 40-70 years, accounting for 41.1% of the male population, which corresponds to the population segment targeted in this study. The total workforce was 3,637,298 people, based on data from the 2012 Economic Census as cited in the same statistical yearbook. Of these, 901,724 (24.8%) were engaged in manufacturing, of whom 292,717 (32.5% of the manufacturing sector) worked in the transport equipment manufacturing industry, including the automobile industry. This was followed by wholesale and retail trade (719,814 workers; 19.8%) and accommodation and food services (346,983 workers; 9.5%). These industrial statistics were based on data collected as of February 1, 2012, and include both men and women. These figures highlight the region’s strong industrial base, particularly its concentration in automobile manufacturing ^(15)^.

### Participant selection

Men who received a health checkup in 2013 were initially identified. Participants were selected based on the following criteria: 1) aged 40 to 70 years at the time of the 2013 checkup, which was intended to ensure that they met the eligibility criteria for Specific Health Checkups in both 2013 and 2017; 2) had data from the 2013 questionnaire on stages of behavioral change; and 3) had no history of lifestyle-related diseases. A history of lifestyle-related diseases was defined by medication use for conditions such as diabetes, hypertension, or dyslipidemia, and a history of stroke (including cerebral hemorrhage and brain infarction), heart disease (such as angina pectoris or myocardial infarction), or chronic kidney disease/kidney failure, as well as receipt of treatment (e.g., dialysis therapy). Individuals with missing data on these variables were excluded. Participants lacking 2017 health checkup data or data required to calculate the propensity score were also excluded. Participants were then divided into two groups based on the median age and analyzed separately.

### Behavioral change stages and outcomes

#### Behavioral change stages

The standard questionnaire in the Japanese Specific Health Checkups includes the question, “Do you want to improve your life habits related to eating and exercising?” ^(16)^ The possible responses are: 1) Do not want to; 2) Want to (within six months); 3) Want to improve soon (within a month) and have started; 4) Already trying to improve (for less than six months); and 5) Already trying to improve (for over six months). These responses correspond to the five stages of behavior change in the TTM: precontemplation, contemplation, preparation, action, and maintenance, respectively ^(2)^. We divided the participants into two groups based on their behavior change stage in 2013: 1) the precontemplation group (those uninterested in improving lifestyle habits), and 2) the contemplation or higher group (those interested in or actively trying to improve their habits).

#### Outcomes

The following exploratory outcomes were assessed based on the 2017 Specific Health Checkups and lifestyle habits from questionnaires: BMI, waist circumference, systolic blood pressure (SBP), diastolic blood pressure (DBP), fasting blood glucose, triglycerides, low-density lipoprotein (LDL), high-density lipoprotein (HDL), aspartate aminotransferase (AST), alanine aminotransferase (ALT), gamma-glutamyltransferase (γ-GT), and various lifestyle habits. The lifestyle habits assessed through the questionnaires included eating speed (quicker than others, normal, slower); eating supper within two hours before bedtime more than three times a week (yes or missing); skipping breakfast more than three times a week (yes or missing); exercising to sweat lightly for over 30 minutes per session at least twice a week for more than a year (yes or missing); walking or engaging in equivalent physical activity for more than one hour per day (yes or missing); walking speed faster than others of the same age and sex (yes or missing); smoking history (currently a heavy smoker, defined as someone who has smoked over 100 cigarettes or has smoked for over six months and continued smoking in the past month) (yes or missing); frequency of alcohol consumption (none or rarely, occasional, or every day); sleeping well and sufficiently (yes or missing); and weight change since the age of 20 (yes or missing). Several lifestyle habits were assessed using questionnaires ^(17)^. For certain items (e.g., exercise habits, eating behaviors), only “yes” responses were explicitly recorded, while non-responses could indicate either a “no” response or missing data. Thus, non-responses were categorized as “missing,” and it was not possible to distinguish between true “no” responses and lack of response.

Participants were categorized into the “precontemplation group,” for those lacking intention to modify their lifestyle habits, or the “contemplation or higher group,” for those who had begun considering changes or were actively pursuing healthier behaviors, based on questionnaire responses about lifestyle habits. These stages correspond to the precontemplation and contemplation-or-higher stages in the TTM and were grouped to reflect a practical distinction between individuals with no behavioral intention and those with at least some readiness for change. We then evaluated potential predictors of these stages of behavior change, including participant characteristics such as age, health conditions, and lifestyle habits. Health conditions were assessed using BMI, waist circumference, SBP, DBP, and blood test results (fasting blood glucose, triglycerides, HDL, LDL, AST, ALT, γ-GT, red blood cell (RBC), hemoglobin (Hb), hematocrit (Ht), and creatinine [CRE]). Lifestyle habits included eating speed, eating before bedtime, skipping breakfast, exercising, physical activity, walking speed, smoking history, frequency of alcohol consumption, sleep patterns, and weight change since age 20.

### Statistical analysis

To assess differences in outcomes between the precontemplation group and the contemplation or higher group, we employed PSM to reduce potential confounding factors and enhance comparability ^(18)^. Given the broad age range (40-70 years), participants were stratified by the median age to adjust for potential age-related variations in health outcomes. A multivariable logistic regression model with generalized estimating equations was employed to calculate the propensity score (PS) while accounting for clustering effects. The following variables from the 2013 health checkup were included as predictors: age; SBP and DBP (mmHg); fasting plasma glucose (mg/dL); triglycerides (mg/dL); HDL (mg/dL); LDL (mg/dL); AST (U/L); ALT (U/L); γ-GT (U/L); RBC (×10^4^/μL); Hb (g/dL); Ht (%); CRE(mg/dL); BMI (kg/m^2^); and abdominal circumference (cm). Additionally, responses from the 2013 lifestyle questionnaire were incorporated, covering factors such as eating speed, exercise habits, smoking, alcohol consumption, and weight change since age 20.

All variables listed in Table 1 were utilized as predictor variables in the PSM process. One-to-one nearest neighbor matching without replacement was performed using a caliper width of 0.2 of the standard deviation of the PS to limit the maximum allowable distance between matched pairs ^(19), (20)^. Performance was assessed by comparing baseline characteristics before and after PSM using standardized differences, with a standardized difference of <0.1 indicating negligible imbalance ^(20)^. PSM was conducted using the STATA module PSMATCH2 developed by Leuven and Sianesi ^(21)^. Categorical and continuous outcomes were analyzed using χ^2^ tests and Student’s *t*-tests, respectively. A significance level of p < 0.05 was applied. All statistical analyses were performed using Stata v18 (StataCorp, College Station, TX, USA).

**Table 1.** Baseline Characteristics from Specific Health Checkups in 2013, Including Laboratory Tests and Questionnaire Items Before Propensity Score Matching.

	Age < 49 years					Age ≥ 49 years				
	(n=5409)					(n=5403)				
	Precontemplation group		Contemplation-or-Higher Stage		Standardized difference	Precontemplation stage		Contemplation-or-Higher Stage		Standardized difference
	(n=1553)		(n=3856)			(n=1830)		(n=3573)		
	mean	SD	mean	SD		mean	SD	mean	SD	
Age (years)	43.6	2.5	43.6	2.5	0.006	56.2	5.3	55.6	5.2	-0.113
SBP (mmHg)	114.9	15.6	117.4	16.4	0.153	120.4	19.3	122.0	18.2	0.087
DBP (mmHg)	73.3	11.5	75.2	12.1	0.156	77.6	12.6	78.9	12.0	0.105
Fasting Plasma Glucose (mg/dL)	98.4	15.0	99.7	16.6	0.086	100.8	16.8	103.2	18.1	0.134
Triglycerides (mg/dL)	117.4	95.6	131.4	101.8	0.142	119.7	105.3	128.9	91.7	0.093
HDL Cholesterol (mg/dL)	59.5	14.1	57.1	13.3	-0.175	61.5	15.0	59.0	13.8	-0.171
LDL Cholesterol (mg/dL)	124.1	30.8	130.9	30.0	0.225	126.3	29.0	131.6	29.6	0.180
AST (U/L)	22.1	11.8	23.7	10.5	0.143	22.1	8.2	23.3	9.8	0.123
ALT (U/L)	25.7	17.8	30.1	20.5	0.228	22.3	13.0	25.6	17.8	0.211
γ-GT (U/L)	41.1	40.1	47.3	50.1	0.136	46.3	53.5	48.8	50.8	0.049
RBC (×10^4^/μL)	498.4	38.2	503.1	36.1	0.126	481.9	39.4	489.4	38.0	0.194
Hb (g/dL)	15.3	0.9	15.3	0.9	0.081	15.0	1.0	15.1	1.0	0.138
Ht (%)	44.6	2.6	44.8	2.5	0.047	44.0	2.8	44.3	2.7	0.128
CRE (mg/dL)	0.8	0.1	0.8	0.1	0.159	0.8	0.1	0.9	0.1	0.103
BMI (kg/m^2^)	22.7	3.5	24.1	3.4	0.387	22.4	2.8	23.6	2.9	0.403
Waist Circumference (cm)	80.7	9.1	84.2	9.0	0.381	81.2	8.0	84.1	7.9	0.368
	**n**	**%**	**n**	**%**		**n**	**%**	**n**	**%**	
Smoking History	728	46.9	1374	35.6	-0.230	783	42.8	1113	31.2	-0.243
Weight Gain ≥10 kg Since Age 20	538	34.6	1845	47.9	0.271	647	35.4	1718	48.1	0.260
Regular Exercise (≥30 min, ≥2×/week, ≥1 year)	210	13.5	677	17.6	0.112	314	17.2	801	22.4	0.132
Daily Walking/Physical Activity (>1 hour)	258	16.6	605	15.7	-0.025	390	21.3	652	18.3	-0.077
Walking Faster Than Peers of the Same Age and Sex	397	25.6	1145	29.7	0.092	581	31.8	1332	37.3	0.117
Eating Speed					-0.092					-0.156
Quicker	627	40.4	1756	45.5		592	32.4	1428	40.0	
Normal	841	54.2	1896	49.2		1079	59.0	1895	53.0	
Slower	85	5.5	204	5.3		159	8.7	250	7.0	
Late Supper within 2 Hours of Bedtime (≥3 times/week)	613	39.5	1658	43.0	0.072	558	30.5	1167	32.7	0.047
Skipping Breakfast (≥3 times/week)	431	27.8	950	24.6	-0.071	307	16.8	475	13.3	-0.098
Alcohol Consumption Frequency					-0.026					-0.048
None or rarely	501	32.3	1097	28.5		527	28.8	935	26.2	
Occasional	465	29.9	1529	39.7		433	23.7	1177	32.9	
Every day	587	37.8	1230	31.9		870	47.5	1461	40.9	
Adequate Sleep	565	36.4	1301	33.7	-0.055	749	40.9	1418	39.7	-0.025

γ-GT: gamma-glutamyltransferase; ALT: alanine aminotransferase; AST: aspartate aminotransferase; BMI: body mass index; CRE: creatinine; DBP: diastolic blood pressure; Hb: hemoglobin; HDL: high-density lipoprotein; Ht: hematocrit; LDL: low-density lipoprotein; RBC: red blood cell count; SBP: systolic blood pressure.

## Results

### Participant characteristics

We identified 28,778 men who received a health checkup in 2013. Of these, 23,316 were aged 40-70 and met the age inclusion criterion. Among them, 23,178 participated in the stages of behavioral change questionnaire. Participants with a history of treatment for lifestyle-related diseases, including high blood pressure, diabetes, dyslipidemia, or other relevant conditions, were excluded (n = 5,759). Individuals with missing data on treatment history or medication use were also excluded (n = 384). After applying the exclusion criteria, 17,035 participants remained eligible for follow-up in 2017. We excluded 5,937 individuals who did not undergo a health checkup in 2017, reducing the sample to 11,098 participants. Additionally, those with missing data required for the PS calculation were excluded (n = 286). The final analysis included 10,812 participants. Their median age was 48 years, and we stratified them into two groups: those under 49 years and those 49 years or older.

Table 1 summarizes the baseline characteristics of the participants included in the final analysis before PSM. Among participants under 49 years (n = 5,409), 1,553 were in the precontemplation group. For participants aged 49 years or older (n = 5,403), 1,830 were in the precontemplation group. Across both age groups, participants in the precontemplation group were less likely to engage in healthier behaviors than those in the contemplation or higher group, exhibiting a higher smoking rate. However, those in the latter groups tended to have poorer health outcomes, including higher BMI and waist circumference, as well as generally worse blood pressure and test results.

### Propensity score-matched participants and adjusted outcomes

PSM identified 1,495 matched pairs for participants under 49 years and 1,749 matched pairs for participants aged 49 years or older. The standardized differences between the groups were below 0.1 for all variables. Table 2 presents the detailed results of blood tests and lifestyle habits assessed through questionnaires after PSM. Table 3 shows the proportions of each stage of behavioral change for both age groups before and after matching. No significant changes were observed in the contemplation or higher group breakdown.

**Table 2.** Baseline Characteristics from Specific Health Checkups in 2013, Including Laboratory Tests and Questionnaire Items after Propensity Score Matching.

	Age < 49 years					Age ≥ 49 years				
	Precontemplation stage		Contemplation-or-Higher Stage		Standardized difference	Precontemplation stage		Contemplation-or-Higher Stage		Standardized difference
	(n=1495)		(n=1495)			(n=1749)		(n=1749)		
	mean	SD	mean	SD		mean	SD	mean	SD	
Age (years)	43.6	2.5	43.6	2.6	-0.014	56.1	5.3	56.4	5.5	0.050
SBP (mmHg)	115.1	15.6	114.6	15.3	-0.038	120.6	19.3	120.4	18.1	-0.009
DBP (mmHg)	73.5	11.5	73.0	11.0	-0.043	77.7	12.6	77.4	11.9	-0.023
Fasting Plasma Glucose (mg/dL)	98.4	15.2	98.2	15.4	-0.019	101.1	17.1	101.0	15.3	-0.003
Triglycerides (mg/dL)	118.7	96.8	116.9	84.1	-0.020	121.1	106.9	119.6	87.5	-0.016
HDL Cholesterol (mg/dL)	59.3	14.2	59.8	14.5	0.037	61.1	14.8	61.6	14.7	0.038
LDL Cholesterol (mg/dL)	125.0	30.7	123.3	28.7	-0.054	127.3	28.8	125.7	29.2	-0.056
AST (U/L)	22.2	11.9	21.8	8.0	-0.044	22.2	8.2	22.1	7.7	-0.012
ALT (U/L)	26.1	17.9	24.9	14.3	-0.075	22.6	13.1	22.0	11.6	-0.049
γ-GT (U/L)	41.5	40.5	40.7	43.0	-0.020	46.6	54.0	46.4	50.7	-0.004
RBC (×10^4^/μL)	499.1	38.2	497.8	34.8	-0.035	483.2	38.5	480.6	38.7	-0.067
Hb (g/dL)	15.3	0.9	15.3	0.9	-0.024	15.0	1.0	15.0	1.0	-0.049
Ht (%)	44.7	2.6	44.6	2.5	-0.014	44.0	2.7	43.9	2.7	-0.050
CRE (mg/dL)	0.8	0.1	0.8	0.1	-0.043	0.8	0.1	0.8	0.1	-0.032
BMI (kg/m^2^)	22.9	3.5	22.6	2.9	-0.080	22.6	2.8	22.3	2.5	-0.099
Waist Circumference (cm)	81.1	9.1	80.4	7.8	-0.079	81.6	7.9	81.0	7.2	-0.075
	**n**	**%**	**n**	**%**		**n**	**%**	**n**	**%**	
Smoking History	676	45.2	735	49.2	0.079	716	40.9	756	43.2	0.046
Weight Gain ≥10 kg Since Age 20	536	35.9	491	32.8	-0.063	641	36.7	580	33.2	-0.073
Regular Exercise (≥30 min, ≥2×/week, ≥1 year)	208	13.9	185	12.4	-0.046	313	17.9	303	17.3	-0.015
Daily Walking/Physical Activity (>1 hour)	247	16.5	273	18.3	0.046	365	20.9	396	22.6	0.043
Walking Faster Than Peers of the Same Age and Sex	392	26.2	356	23.8	-0.056	568	32.5	550	31.5	-0.022
Eating Speed					0.041					0.023
Quicker	613	41.0	582	38.9		581	33.2	553	31.6	
Normal	800	53.5	826	55.3		1018	58.2	1050	60.0	
Slower	82	5.5	87	5.8		150	8.6	146	8.4	
Late Supper within 2 Hours of Bedtime (≥3 times/week)	596	39.9	557	37.3	-0.054	534	30.5	517	29.6	-0.021
Skipping Breakfast (≥3 times/week)	402	26.9	413	27.6	0.017	285	16.3	291	16.6	0.009
Alcohol Consumption Frequency					-0.008					0.030
None or rarely	473	31.6	509	34.1		512	29.3	518	29.6	
Occasional	464	31.0	402	26.9		429	24.5	372	21.3	
Every day	558	37.3	584	39.1		808	46.2	859	49.1	
Adequate Sleep	542	36.3	557	37.3	0.021	719	41.1	716	40.9	-0.003

γ-GT: gamma-glutamyltransferase; ALT: alanine aminotransferase; AST: aspartate aminotransferase; BMI: body mass index; CRE: creatinine; DBP: diastolic blood pressure; Hb: hemoglobin; HDL: high-density lipoprotein; Ht: hematocrit; LDL: low-density lipoprotein; RBC: red blood cell count; SBP: systolic blood pressure.

**Table 3.** Distribution of Precontemplation and Contemplation-or-higher Groups, Including Subcategories, before and after Propensity Score Matching.

	Age < 49 years								Age ≥ 49 years							
	Before propensity score matching				After propensity score matching				Before propensity score matching				After propensity score matching			
	Precontemplation stage		Contemplation-or-Higher Stage		Precontemplation stage		Contemplation-or-Higher Stage		Precontemplation stage		Contemplation-or-Higher Stage		Precontemplation stage		Contemplation-or-Higher Stage	
	(n=1553)		(n=3856)		(n=1495)		(n=1495)		(n=1830)		(n=3573)		(n=1749)		(n=1749)	
	n	%	n	%	n	%	n	%	n	%	n	%	n	%	n	%
Precontemplation	1553	100.0			1495	100.0			1830	100.0			1749	100.0
Contemplation			1830	47.5			735	49.2			1480	41.4			758	43.3
Preparation			756	19.6			290	19.4			672	18.8			335	19.2
Action			547	14.2			203	13.6			420	11.8			184	10.5
Maintenance			723	18.8			267	17.9			1001	28.0			472	27.0

Table 4 presents the outcomes for the matched participants. Among those under 49 years, only HDL and CRE levels showed statistically significant differences (*p* < 0.05). In participants aged 49 years or older, significant differences were observed in HDL, BMI, the proportion of individuals receiving dyslipidemia medications, and eating speed.

**Table 4.** Health Checkup Data and Questionnaire Responses from 2017 after Propensity Score Matching.

	Age < 49 years							Age ≥ 49 years						
	Precontemplation stage			Contemplation-or-Higher Stage			p-value	Precontemplation stage			Contemplation-or-Higher Stage			p-value
	N (total)	mean	SD	N (total)	mean	SD		N (total)	mean	SD	N (total)	mean	SD	
SBP (mmHg)	1493	116.0	17.0	1492	115.8	16.2	0.730	1744	122.5	19.2	1749	121.6	18.4	0.141
DBP (mmHg)	1493	75.3	12.2	1492	75.1	12.0	0.722	1744	78.6	12.1	1749	78.0	11.4	0.117
Fasting Plasma Glucose (mg/dL)	1483	98.6	14.8	1479	98.1	13.9	0.352	1723	101.0	15.2	1747	100.9	14.3	0.813
Triglycerides (mg/dL)	1494	119.7	103.3	1492	118.0	84.6	0.625	1744	115.2	84.9	1749	113.1	73.8	0.428
HDL Cholesterol (mg/dL)	1494	58.7	14.1	1492	59.8	14.9	0.041	1744	61.1	15.1	1749	62.2	14.8	0.034
LDL Cholesterol (mg/dL)	1494	131.2	31.8	1492	131.1	30.5	0.885	1744	131.3	29.9	1749	130.6	30.3	0.495
AST (U/L)	1493	21.7	9.7	1492	21.7	9.1	0.960	1744	22.0	9.7	1749	21.9	9.1	0.770
ALT (U/L)	1493	25.7	16.8	1492	25.2	15.9	0.380	1744	22.1	13.8	1749	21.7	12.4	0.378
γ-GT (U/L)	1493	44.6	47.3	1492	44.2	44.4	0.795	1744	45.4	55.5	1749	45.1	59.6	0.901
CRE (mg/dL)	1494	0.8	0.1	1492	0.8	0.1	0.049	1744	0.9	0.2	1748	0.9	0.2	0.647
BMI (kg/m^2^)	1493	23.2	3.5	1492	23.0	3.0	0.152	1744	22.7	2.9	1749	22.5	2.6	0.014
Waist Circumference (cm)	1487	82.4	9.2	1483	82.0	8.1	0.191	1725	82.5	8.2	1721	82.1	7.5	0.095
	**N (total)**	**n**	**%**	**N (total)**	**n**	**%**	**p-value**	**N (total)**	**n**	**%**	**N (total)**	**n**	**%**	**p-value**
Smoking History	1486	639	43.0	1483	666	44.9	0.295	1724	646	37.5	1720	641	37.3	0.902
Medications for Hypertension	1486	49	3.3	1483	48	3.2	0.926	1724	147	8.5	1720	154	9.0	0.657
Medications for Diabetes	1486	20	1.4	1483	24	1.6	0.539	1724	35	2.0	1720	36	2.1	0.897
Medications Dyslipidemia	1486	24	1.6	1483	35	2.4	0.146	1724	42	2.4	1720	67	3.9	0.014
History of Stroke	1486	3	0.2	1483	2	0.1	0.656	1724	10	0.6	1720	9	0.5	0.822
History of Heart Disease	1486	8	0.5	1483	13	0.9	0.271	1724	37	2.2	1720	41	2.4	0.639
History of Chronic Kidney Disease/Kidney Failure	1486	0	0.0	1483	1	0.1	0.317	1724	1	0.1	1723	1	0.1	0.999
Weight Gain ≥10 kg Since Age 20	1486	588	39.3	1495	563	37.7	0.347	1749	661	37.8	1749	633	36.2	0.327
Regular Exercise (≥30 min, ≥2×/week, ≥1 year)	1495	216	14.5	1495	229	15.3	0.504	1749	308	17.6	1749	349	20.0	0.076
Daily Walking/Physical Activity (>1 hour)	1495	261	17.5	1495	272	18.2	0.599	1749	352	20.1	1749	361	20.6	0.706
Walking Faster Than Peers of the Same Age and Sex	1495	404	27.0	1495	435	29.1	0.207	1749	566	32.4	1749	616	35.2	0.074
Eating Speed	1485			1489			0.534	1740			1739			0.049
Quicker		791	53.3		822	55.2			992	57.0		1051	60.4	
Normal		608	40.9		580	39.0			567	32.6		542	31.2	
Slower		86	5.8		87	5.8			181	10.4		146	8.4	
Late Supper within 2 Hours of Bedtime (≥3 times/week)	1495	567	37.9	1495	538	36.0	0.272	1749	484	27.7	1749	438	25.0	0.078
Skipping Breakfast (≥3 times/week)	1495	392	26.2	1495	375	25.1	0.477	1749	260	14.9	1749	253	14.5	0.738
Alcohol Consumption Frequency	1489			1487			0.990	1744			1747			0.759
None or rarely		467	31.4		469	31.5			521	29.9		532	30.5	
Occasional		452	30.4		448	30.1			441	25.3		423	24.2	
Every day		570	38.3		570	38.3			782	44.8		792	45.3	
Adequate Sleep	1495	559	37.4	1495	538	36.0	0.426	1749	767	43.9	1749	716	40.9	0.081

γ-GT: gamma-glutamyltransferase; ALT: alanine aminotransferase; AST: aspartate aminotransferase; BMI: body mass index; CRE: creatinine; DBP: diastolic blood pressure; Hb: hemoglobin; HDL: high-density lipoprotein; Ht: hematocrit; LDL: low-density lipoprotein; RBC: red blood cell count; SBP: systolic blood pressure.

## Discussion

We assessed differences in health status and behaviors four years later based on participants’ baseline stage of behavioral change, differentiating between those in the precontemplation or unmotivated stage and those in the contemplation or later stages, using data from the Specific Health Checkups. Of the 28 outcome measures, two in men under 49 years and four in men aged 49 years or older showed statistically significant differences (p < 0.05).

While these findings are significant, they should be interpreted with caution. This exploratory study aimed to identify a broad spectrum of potential differences in health outcomes and behaviors, rather than test specific hypotheses. The large number of outcomes analyzed increases the risk of type I error (α error), and some observed differences may reflect random variation rather than true associations. No adjustments for multiple comparisons were made, as such adjustments may obscure true associations in exploratory analyses ^(22)^. Caution is advised in interpreting *p*-values, particularly when analyzing a large number of outcomes, to avoid overestimating the significance of differences ^(23)^. The lack of significant differences in long-term health behaviors between the precontemplation and contemplation or later stages may reflect the cyclical and nonlinear nature of behavior change, as the TTM describes ^(3)^. This model views behavior changes as a dynamic process in which individuals can progress, regress, or repeat stages over time, highlighting the need for sustained support to achieve lasting improvements.

Previous studies have reported that individuals in the contemplation or later stages often have higher rates of comorbidities, such as hypertension and diabetes, which may increase their awareness of the need for behavioral improvement ^(24)^. This heightened awareness may result in regression to the mean, where individuals with initially poor health metrics tend to show natural improvements over time, regardless of intentional lifestyle changes ^(10)^. Conversely, individuals in the precontemplation stage, who often have better baseline health, may experience less noticeable changes over time, which could mask differences between groups ^(10), (24)^.

Additionally, while higher motivational stages are associated with short-term behavior changes, the effect may diminish over time without sustained support. Studies have shown that initial improvements in behaviors like smoking cessation or weight loss often relapse within the first year, particularly when external support diminishes ^(25), (26)^. Similarly, physical activity interventions typically show maintenance for up to six months, but activity levels may decrease or cease without further reinforcement or follow-up ^(27)^. This aligns with research on physical activity adoption, emphasizing the need for structured and ongoing support ^(28)^. These findings also parallel studies on weight management, where weight loss achieved through lifestyle modifications is often followed by weight regain in the absence of continuous intervention ^(29)^. Lifestyle modifications have proven beneficial in managing metabolic syndrome and improving health outcomes; however, maintaining these improvements requires continued psychological support, such as motivational interviewing and behavioral change therapy, to ensure long-term success ^(30)^.

Our findings highlight the unique challenges faced by men in the precontemplation stage. These individuals often lack health awareness, psychological readiness, and active engagement in health-related behaviors ^(31)^. In this stage, traditional motivational factors such as decisional balance and self-efficacy, central to the TTM, may be less immediately relevant. Instead, increasing health awareness is a critical foundational step to help individuals recognize the risks associated with their current behaviors ^(31)^. This approach is supported by prior research, which emphasizes the importance of enhancing psychological readiness and promoting simple gateway behaviors to encourage initial health-related actions ^(3), (31)^. Overcoming these barriers allows stage-specific interventions to better support men in the precontemplation stage by fostering behavior change and improving long-term health outcomes. This highlights the importance of tailoring interventions to match individuals’ needs and readiness at different stages of change.

Given the varying levels of motivation and readiness, our findings underscore the need for personalized health interventions. Designing stage-specific strategies that consider individual readiness, psychological needs, and contextual factors could enhance the effectiveness of health guidance, particularly for those in the precontemplation stage. Tailored approaches, such as targeted health education or motivational interviewing, are essential to address individual barriers and promote sustained behavioral change. These interventions align with the TTM principles and support broader public health goals by facilitating more meaningful and lasting improvements in health outcomes.

This study has several limitations. First, it relied on health checkup data from a single region in Japan, which introduces the possibility of selection bias and limits the generalizability of our findings to broader populations. Second, while PSM reduces confounding, it cannot fully eliminate bias from unmeasured or unknown factors, as would be possible in a randomized controlled trial. Thus, residual confounding may still influence the results. Furthermore, the observational nature of this study precludes causal inference between behavioral stages and long-term health outcomes. Third, although occupational categories were available in the dataset, the information was collected by the health screening organization for clinical reference purposes rather than for research, and participants were allowed to select multiple job types. Moreover, no information was available on company size or employment status. Therefore, we did not include occupation-related variables in our analysis.

Future research should expand on these findings by evaluating the effectiveness of stage-specific interventions in longitudinal and randomized controlled studies. Investigating the interplay between motivational factors, environmental supports, and social determinants of health could provide valuable insights into strategies for promoting sustainable health behavior change. In addition, our findings suggest that applying the TTM at the population level may obscure important individual differences in motivation. For example, the precontemplation stage may include both individuals who are indifferent to health because they are currently healthy, and others who are unaware of their own health risks despite needing behavioral change. This heterogeneity complicates interpretation and may dilute associations with outcomes. Therefore, rather than using the TTM to justify uniform interventions based on stage categorization, it should be employed as a tool to help assess individual readiness for change and guide personalized interventions. This approach may improve the effectiveness of health guidance programs in practice.

In conclusion, using Japanese data from Specific Health Checkups and PSM, this study found no consistent association between behavioral change stages and health behaviors or clinical outcomes among middle-aged and older Japanese men.

## Article Information

### Acknowledgements

The authors are grateful to Naoyoshi Kariya, Hidehito Tanaka, and Yoshihiro Tsutsumi for their valuable advice on the use of the Aichi Health Promotion Study dataset. We also sincerely thank Masahiro Matsunaga, Tomohiro Umemura, Takashi Kawagoe, Eiji Shibata, and Reiko Hori for their insightful contributions to the discussion. Finally, we would like to acknowledge Editage (www.editage.com) for providing editing services for the English language.

### Author Contributions

Rei Wakayama conceptualized the study, managed the research funding, extracted the necessary data from the dataset, conducted the data analysis, and drafted the manuscript. Akihiko Narisada provided and managed the dataset, contributed to data interpretation, and critically reviewed and edited the manuscript. Kohta Suzuki supervised the study, provided guidance and support throughout the research process, and contributed to manuscript revision.

### Conflicts of Interest

None

### Approval code from IR

This study was approved by the Institutional Review Board (IRB) of Aichi Medical University School of Medicine (Approval Number: 18-M005).

### Data Availability

The data used in this study were provided by the Aichi Health Promotion Foundation under license and are not publicly available. Access to these data requires permission from the foundation. Researchers may contact the corresponding author for further details.

## References

[1] Kohro T, Furui Y, Mitsutake N, et al. The Japanese national health screening and intervention program aimed at preventing worsening of the metabolic syndrome. Int Heart J. 2008;49(2):193-203. 18475019 10.1536/ihj.49.193

[2] Prochaska JO, DiClemente CC. Transtheoretical therapy: toward a more integrative model of change. Psychotherapy. 1982;19(3):276-88.

[3] Nutbeam D, Harris E, Wise M. Theory in a nutshell: a practical guide to health promotion theories. 4th ed. New York: McGraw-Hill; 2021. p. 12-4.

[4] The National Health and Nutrition Survey in Japan [Internet]. Ministry of Health, Labour and Welfare. 2019 [cited 2024 Dec 6]. Available from: https://www.mhlw.go.jp/content/000711008.pdf

[5] Material No. 4. Materials of the 2nd meeting of the Headquarters for Social Security Reform and Work Style Reform Toward 2040 [Internet]. Ministry of Health, Labour and Welfare. 2019 [cited 2024 Dec 6]. Available from: https://www.mhlw.go.jp/content/12601000/000514142.pdf

[6] Material No. 3-1. Materials of the 16th meeting of the Promotion Expert Committee for Health Japan 21 (the second term): data on healthy life expectancy in 2019 [Internet]. Ministry of Health, Labour and Welfare. 2021 [cited 2024 Dec 6]. Available from: https://www.mhlw.go.jp/content/10904750/000872952.pdf

[7] Prochaska JO, DiClemente CC. Stages and processes of self-change of smoking: toward an integrative model of change. J Consult Clin Psychol. 1983;51(3):390-5. 6863699 10.1037//0022-006x.51.3.390

[8] Riemsma RP, Pattenden J, Bridle C, et al. Systematic review of the effectiveness of stage based interventions to promote smoking cessation. BMJ. 2003;326(7400):1175-7. 12775617 10.1136/bmj.326.7400.1175 PMC156457

[9] Barzegar H, Sodagar S, Seirafi M, et al. Blood pressure management protocol based on transtheoretical model effectiveness on self- care: a systematic review. Health Promot Perspect. 2024;14(3):207-20. 39633620 10.34172/hpp.42814 PMC11612347

[10] Kimura H, Asahi K, Tanaka K, et al. Health-related behavioral changes and incidence of chronic kidney disease: the Japan Specific Health Checkups (J-SHC) Study. Sci Rep. 2022;12(1):16319. 36175537 10.1038/s41598-022-20807-2 PMC9522825

[11] Mizuno A, Kaneko H, Suzuki Y, et al. Enduring relevance of the stages of change model for transforming lifestyle behaviors. Circ J. 2023;87(8):1138-42. 37394571 10.1253/circj.CJ-23-0292

[12] Barnett AG, van der Pols JC, Dobson AJ. Regression to the mean: what it is and how to deal with it. Int J Epidemiol. 2005;34(1):215-20. 15333621 10.1093/ije/dyh299

[13] Artinian NT, Fletcher GF, Mozaffarian D, et al. Interventions to promote physical activity and dietary lifestyle changes for cardiovascular risk factor reduction in adults: a scientific statement from the American Heart Association. Circulation. 2010;122(4):406-41. 20625115 10.1161/CIR.0b013e3181e8edf1 PMC6893884

[14] Rosenbaum PR, Rubin DB. The central role of the propensity score in observational studies for causal effects. Biometrika. 1983;70(1):41-55.

[15] Aichi prefectural statistical yearbook (FY2014) [Internet]. Aichi Prefectural Government. 2014 [cited 2025 Mar 27]. Available from: https://www.pref.aichi.jp/soshiki/toukei/nenkan-2014nd.html

[16] Standard questionnaire items for Specific Health Checkups [Internet]. Ministry of Health, Labour and Welfare. 2024 [cited 2024 Dec 6]. Available from: https://www.mhlw.go.jp/seisakunitsuite/bunya/kenkou_iryou/kenkou/seikatsu/dl/hoken-program2_02.pdf

[17] Manual for smooth implementation of specific health checkups and specific health guidance. ver2.0 [Internet]. Ministry of Health, Labour and Welfare. 2013 [cited 2024 Dec 6]. Available from: https://www.mhlw.go.jp/bunya/shakaihosho/iryouseido01/pdf/info03d-1.pdf

[18] Griswold ME, Localio AR, Mulrow C. Propensity score adjustment with multilevel data: setting your sites on decreasing selection bias. Ann Intern Med. 2010;152(6):393-5. 20231571 10.7326/0003-4819-152-6-201003160-00010

[19] Stuart EA. Matching methods for causal inference: a review and a look forward. Stat Sci. 2010;25(1):1-21. 20871802 10.1214/09-STS313 PMC2943670

[20] Austin PC. An introduction to propensity score methods for reducing the effects of confounding in observational studies. Multivariate Behav Res. 2011;46(3):399-424. 21818162 10.1080/00273171.2011.568786 PMC3144483

[21] Leuven E, Sianesi B. PSMATCH2: Stata module to perform full Mahalanobis and propensity score matching, common support graphing, and covariate imbalance testing; 2018 [Internet]. [cited 2025 Apr 4]. Available from: https://ideas.repec.org/c/boc/bocode/s432001.html

[22] Rothman KJ. No adjustments are needed for multiple comparisons. Epidemiology. 1990;1(1):43-6. 2081237

[23] Wasserstein RL, Lazar NA. The ASA statement on p -values: context, process, and purpose. Am Stat. 2016;70(2):129-33.

[24] Takada D, Kunisawa S, Kikuno A, et al. Stages of a transtheoretical model as predictors of the decline in estimated glomerular filtration rate: a retrospective cohort study. J Epidemiol. 2022;32(7):323-9. 33487611 10.2188/jea.JE20200422 PMC9189319

[25] Wing RR, Hill JO. Successful weight loss maintenance. Annu Rev Nutr. 2001;21(1):323-41. 11375440 10.1146/annurev.nutr.21.1.323

[26] Howlett N, Trivedi D, Troop NA, et al. Are physical activity interventions for healthy inactive adults effective in promoting behavior change and maintenance, and which behavior change techniques are effective? A systematic review and meta-analysis. Transl Behav Med. 2019;9(1):147-57. 29506209 10.1093/tbm/iby010 PMC6305562

[27] Marcus BH, Dubbert PM, Forsyth LH, et al. Physical activity behavior change: issues in adoption and maintenance. Health Psychol. 2000;19(1S):32-41. 10709946 10.1037/0278-6133.19.suppl1.32

[28] Tuah NA, Amiel C, Qureshi S, et al. Transtheoretical model for dietary and physical exercise modification in weight loss management for overweight and obese adults. Cochrane Database Syst Rev. 2011;(10):CD008066. 21975777 10.1002/14651858.CD008066.pub2

[29] García-Rodríguez O, Secades-Villa R, Flórez-Salamanca L, et al. Probability and predictors of relapse to smoking: results of the National Epidemiologic Survey on Alcohol and Related Conditions (NESARC). Drug Alcohol Depend. 2013;132(3):479-85. 23570817 10.1016/j.drugalcdep.2013.03.008 PMC3723776

[30] Shimazaki T, Okoshi H, Yamauchi T, et al. The process of behavioral change in individuals who are uninterested in health: a qualitative study based on professional health knowledge. Environ Health Prev Med. 2022;27:32. 35896370 10.1265/ehpm.22-00072 PMC9357552

[31] Bassi N, Karagodin I, Wang S, et al. Lifestyle modification for metabolic syndrome: a systematic review. Am J Med. 2014;127(12):1242.e1-10. 10.1016/j.amjmed.2014.06.035 25004456

